# Continuous Temperature-Monitoring Socks for Home Use in Patients With Diabetes: Observational Study

**DOI:** 10.2196/12460

**Published:** 2018-12-17

**Authors:** Alexander M Reyzelman, Kristopher Koelewyn, Maryam Murphy, Xuening Shen, E Yu, Raji Pillai, Jie Fu, Henk Jan Scholten, Ran Ma

**Affiliations:** 1 California School of Podiatric Medicine Samuel Merritt University San Francisco, CA United States; 2 Northport VA Medical Center Northport, CA United States; 3 Siren Care Inc San Francisco, CA United States; 4 Siren Care (Shanghai) Information Technology Co Ltd Shanghai China; 5 Medical Affairs Consulting Inc Menlo Park, CA United States

**Keywords:** diabetes, diabetic foot ulcer, continuous temperature monitoring, Charcot arthropathy, digital health, wearable, neurofabric, mobile phone, wireless, Bluetooth, neuropathy, home use

## Abstract

**Background:**

Over 30 million people in the United States (over 9%) have been diagnosed with diabetes. About 25% of people with diabetes will experience a diabetic foot ulcer (DFU) in their lifetime. Unresolved DFUs may lead to sepsis and are the leading cause of lower-limb amputations. DFU rates can be reduced by screening patients with diabetes to enable risk-based interventions. Skin temperature assessment has been shown to reduce the risk of foot ulceration. While several tools have been developed to measure plantar temperatures, they only measure temperature once a day or are designed for clinic use only. In this report, wireless sensor-embedded socks designed for daily wear are introduced, which perform continuous temperature monitoring of the feet of persons with diabetes in the home environment. Combined with a mobile app, this wearable device informs the wearer about temperature increases in one foot relative to the other, to facilitate early detection of ulcers and timely intervention.

**Objective:**

A pilot study was conducted to assess the accuracy of sensors used in daily wear socks, obtain user feedback on how comfortable sensor-embedded socks were for home use, and examine whether observed temperatures correlated with clinical observations.

**Methods:**

Temperature accuracy of sensors was assessed both prior to incorporation in the socks, as well as in the completed design. The measured temperatures were compared to the reference standard, a high-precision thermostatic water bath in the range 20°C-40°C. A total of 35 patients, 18 years of age and older, with diabetic peripheral neuropathy were enrolled in a single-site study conducted under an Institutional Review Board–approved protocol. This study evaluated the usability of the sensor-embedded socks and correlated the observed temperatures with clinical findings.

**Results:**

The temperatures measured by the stand-alone sensors were within 0.2°C of the reference standard. In the sensor-embedded socks, across multiple measurements for each of the six sensors, a high agreement (*R*^2^=1) between temperatures measured and the reference standard was observed. Patients reported that the socks were easy to use and comfortable, ranking them at a median score of 9 or 10 for comfort and ease of use on a 10-point scale. Case studies are presented showing that the temperature differences observed between the feet were consistent with clinical observations.

**Conclusions:**

We report the first use of wireless continuous temperature monitoring for daily wear and home use in patients with diabetes and neuropathy. The wearers found the socks to be no different from standard socks. The temperature studies conducted show that the sensors used in the socks are reliable and accurate at detecting temperature and the findings matched clinical observations. Continuous temperature monitoring is a promising approach as an early warning system for foot ulcers, Charcot foot, and reulceration.

## Introduction

An estimated 30.3 million people in the United States (ie, 9.4% of the US population) have been diagnosed with diabetes, according to the Centers for Disease Control and Prevention [1].

### Complications of Diabetes, Foot Ulcers, and Prognosis

Diabetes damages blood vessels and nerves, particularly in the feet, and can lead to severe infections that are difficult to treat. About 25% of people with diabetes will experience a diabetic foot ulcer (DFU) in their lifetime [2-4]. When circulation is so poor that a foot ulcer fails to heal or when treatment fails to stop the spread of an infection, sepsis can result. In such cases, amputation is often necessary. Diabetes is the leading cause of lower-limb amputations; DFUs precede approximately 84% of nontraumatic major amputations among people with diabetes [5-7]. The rates of recurrence of foot ulcers are very high, being greater than 40% after one year and 60% within three years [2,8].

Charcot foot, also called Charcot arthropathy, is one of the most debilitating outcomes of diabetes [9]. The condition causes increased blood flow to the foot and increased bone resorption. Immediately keeping all weight off, or off‐loading, of incipient Charcot foot appears to minimize fractures and incapacitating deformities. However, there is a potential for delayed diagnosis and therapeutic intervention as plain x‐rays may not show fractures at the early stages [10].

### Cost to the Health Care System

Diabetic foot ulcers result in considerable cost to the health care system when immediate ulcer episodes, social services, home care, and subsequent ulcer episodes are taken into consideration. Patients with a DFU were seen by their outpatient health care provider about 14 times per year and were hospitalized about 1.5 times per year. The cost of care for these patients was substantial, at about US $33,000 for total reimbursement of all Medicare services per year [11].

The total direct and indirect estimated cost of diagnosed diabetes in the United States in 2012 was US $245 billion. After adjusting for age group and sex, average medical expenditures among people with diagnosed diabetes were about 2.3 times higher than expenditures for people without diabetes [12] .

The national inpatient and emergency department bill summed to US $8.78 billion per year, averaging US $115,957 per case for major amputations [13].

### Diagnosing Foot Ulcers

Screening patients with diabetes to identify those at risk for foot ulceration has been shown to be beneficial [3]. DFU rates can be reduced with screening and appropriate intervention [4,14].

Self-care is a critical factor in detecting early signs of ulcers and injury. However, visual inspection has limitations (eg, patients with obesity or visual impairment cannot see their feet easily); hence, it is not very effective to identify the early signs. A recent study using a remote foot-temperature-monitoring system showed the ability to detect 97% of nontraumatic DFUs five weeks before they presented to the participant and/or clinician [15,16]. Additional options for detecting ulcers early in order to treat and heal ulcer wounds successfully may help prevent lower-limb amputations.

In diabetic foot complications such as foot ulcers and osteomyelitis, elevated temperatures in regions of the foot have been shown to be a precursor for ulceration [17]. In Charcot foot cases, increased plantar foot temperature is observed and is strongly correlated with the location of arthropathy. Temperatures decreased in a predictable manner as acute arthropathy resolved [18,19].

Thus, skin temperature assessment in persons with diabetes is a valuable tool for assessing inflammation in diabetic feet, as well as its resolution [20,21]. Home temperature monitoring has been shown to be an effective approach as an early warning system, to provide patients with objective feedback so they can modify their activity and protect their foot before ulcers develop; such monitoring is included in the International Working Group on Diabetic Foot clinical practice guidelines [16,22].

A handheld, infrared, dermal thermometer was designed to take temperatures on the bottom of both feet at six different spots each morning and compare these temperatures from spot to spot. Temperature differences of 4°F (2.22°C) or higher observed at comparable spots between the feet serve as an early sign of DFUs [16,23-25]. However, this tool has shown limited adoption. A reported shortcoming is that the manual temperature measurement on specific spots on the foot is subjective: asymmetric analysis tended to find false abnormal areas when the left and right feet had different sizes and shapes.

Digital health is a vast and burgeoning field and spans several aspects of health management. With the advent of the Internet of Things and the Internet of Medical Things coupled with smart devices, the potential for improved home care for medical applications is fast becoming a reality. Such devices can facilitate the management of chronic conditions at home, including the effective and timely management of DFUs. Diabetic foot scanners and voice-enabled scales are in development [26]. A “smart mat” allows daily measurement of plantar temperature, compares the temperature profile of the two feet, and aims to identify regions with increased temperature, detecting potential ulcer formation at an early stage [15,27].

Innovation in wearables has led to the development of “smart socks,” with embedded sensors for measuring temperature and reporting increases. A recent report describes socks made entirely of optical fiber [28]. While laboratory testing verified the accuracy of the sensors within, the fragility of the optical fiber resulted in limited usability. Another report describes socks that measure temperature at 10-minute intervals and contain wires [29].

### Continuous Temperature Monitoring and Goal of This Study

All the tools described above are designed to measure temperatures once a day or at long intervals, are for clinic use only, or include wired data transmission. Once-a-day measurements present a risk of giving false positives. Continuous monitoring allows the assessment of temperature over longer periods, taking into consideration varying levels of activity over time, and thus has a greater potential to report consistent and clinically relevant temperature increases [30]. Continuous temperature monitoring can reduce false positives and has the potential to further improve home care and early detection.

Here, we introduce wireless sensor-embedded socks, made of neurofabric textile with microsensors embedded directly into the fabric—for continuous temperature monitoring of the feet of people with diabetes—and wireless reporting. They are designed to be easy to use and are washable as well as reusable.

A pilot study was conducted to assess (1) how comfortable sensor-embedded socks were for daily use and (2) whether observed temperatures correlated with clinical observations. Illustrative cases are presented.

## Methods

### Sensor-Embedded Socks

The socks are made of “smart textile”: textile with microsensors woven directly into the fabric (Siren Diabetic Socks, Neurofabric, Siren Care Inc, San Francisco, CA). These virtually invisible sensors are seamlessly integrated into the socks to monitor temperature changes on the bottom of the feet. The sensor-embedded socks are designed to be reusable and are machine washable and dryable.

**Figure 1:**
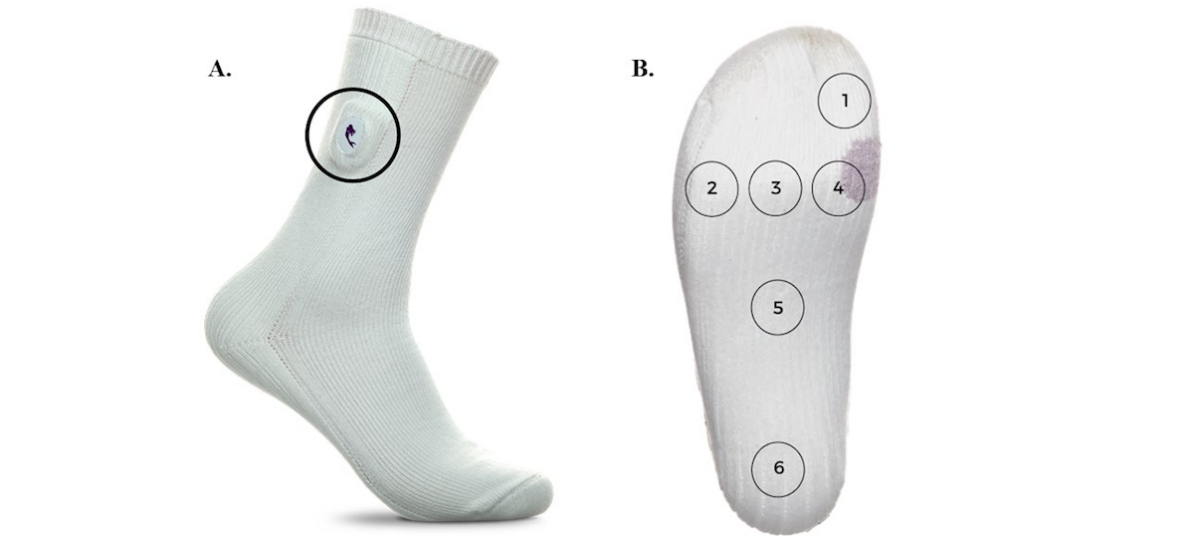
(A) Image of socks with tag (circled) containing battery, microcontroller unit, and Bluetooth chip. (B) Bottom of socks where sensors are located at the hallux (sensor 1), metatarsal points 1,3, and 5 (sensors 2-4), midfoot (sensor 5), and heel (sensor 6).

The sensors embedded in the socks are connected to a small tag on the sock, which encases a microcontroller unit, battery, and Bluetooth chip (see Figure 1A). The six sensors take temperature measurements at 10-second intervals to track temperature increases at the bottom of the user's feet, specifically at the hallux; metatarsal points (MTPs) 1, 3, and 5; midfoot; and heel (see Figure 1B). The data are stored in the tag and sent via Bluetooth to the phone paired with each pair of socks.

**Figure 2:**
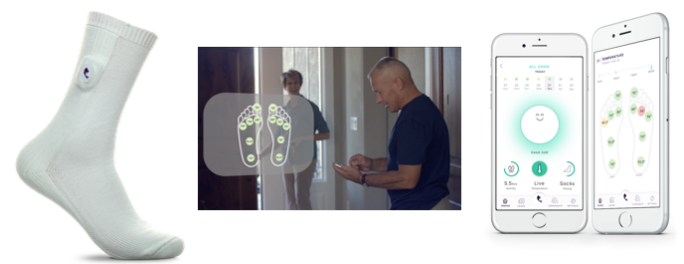
Typical workflow for sensor-embedded socks. The left-hand image shows a temperature-sensing sock: passive continuous monitoring of six key locations occur on the foot. The center image represents continuous monitoring: algorithms monitor temperature reading and generate alerts. The right-hand image displays the patient interface of the app, used for viewing the alerts.

The mobile phone app can be programmed to generate alerts when the user’s feet show temperature increases that could be a warning sign of a potential ulcer (see Figure 2). In this study, the mobile phone app displayed temperature readings to the user, but alerts were not generated.

### Assessment of Accuracy of Sensors Embedded in Socks in Detecting Temperature

Sensors were tested prior to and after incorporation in the socks using a high-precision, thermostatic water bath (Zhejiang Jinbo Electronic Co, Ltd, China) and verified with a 0.01°C high-precision mercury thermometer. The stand-alone sensors were tested at four temperatures: 20°C, 25°C, 37°C, and 45°C.

The sensors woven into the socks were tested in the range 20°C-40°C. Three pairs of socks were tested by immersion in the thermostatic water bath for 10 seconds. The temperature recorded in the sock tag was compared with the reference standard.

### Assessment of Sensor-Embedded Socks Worn by Patients With Diabetes

A single-site study was conducted under an Institutional Review Board-approved protocol to evaluate the usability of the smart socks for patients with diabetic peripheral neuropathy (DPN). Informed consent was obtained from all patients.

A total of 35 patients, 18 years of age and older, from a private clinical practice were enrolled into three groups based on patient-reported medical history and/or medical documents. The groups were as follows: (1) Group 1 included subjects with DPN and no previous history of ulcers (n=11), (2) Group 2 included subjects with DPN and a previous history of ulcers (n=13), and (3) Group 3 included subjects with DPN and a current preulcer as determined by the investigator (n=11).

Subjects participated in two clinic visits. In the first visit, screening procedures were conducted, which included the following: a general physical exam performed by a board-certified podiatrist, visual foot inspection, digital photographs of both feet, and medical history intake. Subjects were provided with the socks and were given an Android mobile phone with the app needed for temperature monitoring. The socks were wirelessly connected with the mobile phone via Bluetooth. The patients were instructed to wear the socks continuously for 6 hours, after which the socks could be removed. The data were streamed via Bluetooth directly to the Android app installed on the phone provided to the patient during the screening and initiation visit. All data were stored in the sock tag and sent via Bluetooth to the phone paired to the socks that were assigned to the enrolled patient.

At the second or end-of-study visit to the clinic—7 days plus or minus 2 days from the screening and initiation visit—the socks were returned to the investigator and the patient was examined for potential adverse reactions. An exit questionnaire was completed to obtain usability information from the patient on the comfortableness of the socks, the ease of Android app use, and the practicality of integrating this specific system into the patient’s everyday life. Upon exit from the trial, data were exported from the Android phone to a secure laptop for analysis. All data collected were deidentified and only subject numbers were used for the duration of the trial.

## Results

### Accuracy of Sensor-Embedded Socks

#### Testing of Sensors not Incorporated in Socks

A total of 36 stand-alone sensors were tested in a high-precision thermostatic water bath for 10 seconds at four temperatures: 20°C, 25°C, 37°C, and 45°C. The results are shown in Table 1. The temperatures measured by the sensors were within 0.2°C of the reference standard, demonstrating the high accuracy of the sensors used in the socks.

**Table 1:** Stand-alone sensor temperature measurements at four water bath temperatures.

Temperature of water bath (reference standard), °C	Stand-alone sensor temperature, °C		
	Mean (SD)	Minimum	Maximum
20	20.08 (0.04)	19.99	20.13
25	25.11 (0.06)	25.00	25.18
37	37.03 (0.07)	36.91	37.10
45	44.92 (0.08)	44.81	45.01

#### Testing of Sensors Embedded in Socks

**Figure 3:**
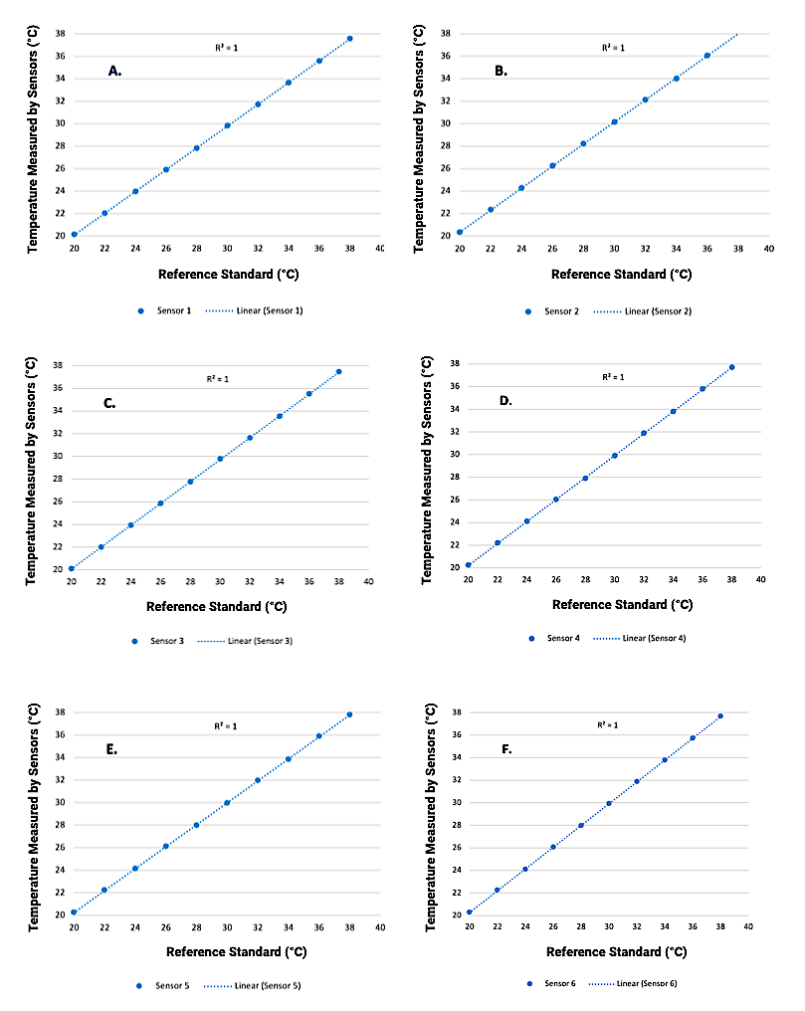
Panels A-F show data for sensors 1-6, positioned at the hallux, metatarsal point 1, metatarsal point 3, metatarsal point 5, arch (midfoot), and heel, respectively. The x-axis shows the reference standard and the y-axis shows the temperatures measured by the sensors embedded in the socks.

Three pairs of socks with embedded sensors were tested by immersion in a thermostatic water bath for 10 seconds, in the range 20°C-40°C. The temperature recorded in the sock tag was compared with the reference standard. The average of 18 measurements for each of six sensors is displayed in Figure 3.

For each of the six sensors, there was a high agreement (*R*^2^=1) between temperatures measured and the reference standard, establishing that sensor-embedded socks can accurately measure temperature across the wide range tested of 20°C-40°C.

### Patient Experience of Sensor-Embedded Socks

#### Patient Information

A total of 35 patients with diabetes assigned to three groups were included in the study as summarized in Table 2.

#### User Experience

**Figure 4:**
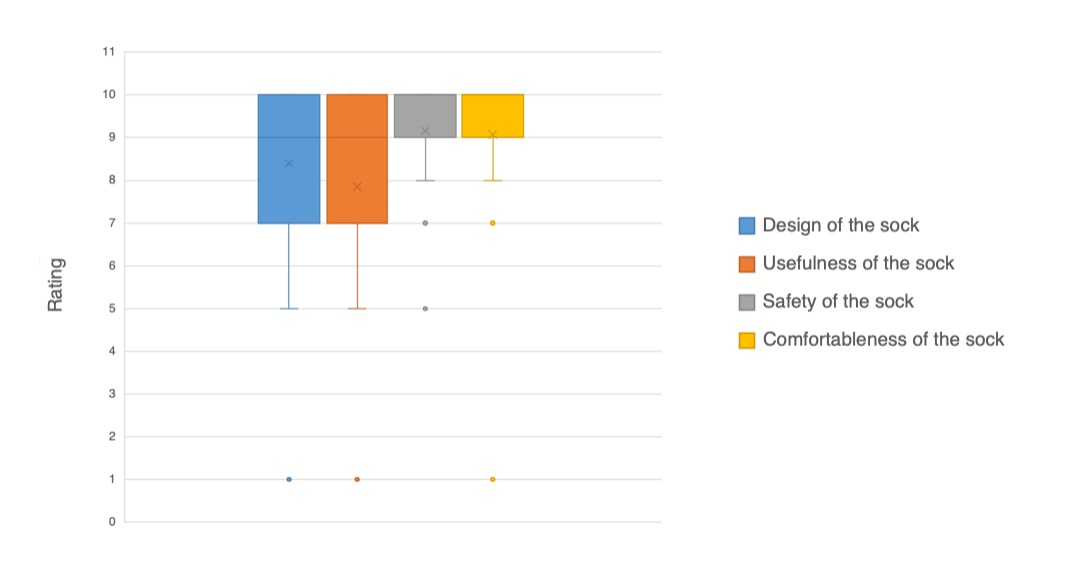
Patients reported on their experience on a scale of 1 to 10 where 10 is “Good” and 1 is “Bad.” In the box and whisker plot, the line within the box represents the median, the x in the box represents the mean, the bounds of the box are at the 1st and 3rd quartiles (25% and 75%), the whisker (vertical line) extends to the minimum value, and the dots are outliers.

Patients wore the socks at home for 3-21 hours (median 7). Some patients wore the socks at night and slept in them. Upon their return visit to the clinic, they returned the socks and provided feedback via the exit questionnaire on different aspects of the socks, such as design, usefulness, and comfortableness. The results are shown in Figure 4. In these patients’ experience while wearing the socks at home, they found the socks to be safe, comfortable, useful, and well-designed. For each question, the median response was 9 or 10 on a 10-point scale, indicating a high level of satisfaction.

**Figure 5:**
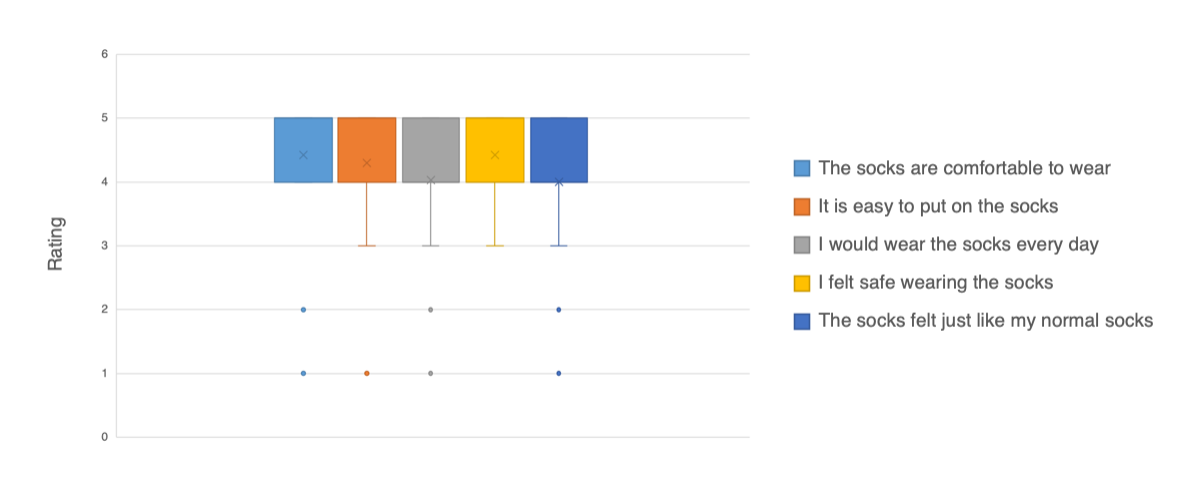
Patients reported on their experience on a scale of 1-5, where 5 is “Completely Agree” and 1 is “Completely Disagree.” In each box and whisker plot, the line within the box represents the median, the x in the box represents the mean, the bounds of the box are at the 1st and 3rd quartiles (25% and 75%), the whisker (vertical line) extends to the minimum values, and the dots are outliers.

Patients also provided feedback on a different scale for a separate set of questions (see Figure 5), some overlapping with the earlier questions. The findings were consistent with those from the 10-point scale. The median value for each of the responses was 4 or 5 on a 5-point scale, confirming a positive experience. Patients reported that the socks felt just like their normal, everyday socks. Notably, patients did not feel they were wearing sensor-embedded socks or that they had to use the socks carefully. Their stated willingness to wear the socks every day underscores the socks’ suitability for home use. This suggests that smart textiles, which are used to make the sensor-embedded socks, can seamlessly integrate into the life of the wearer and are suitable for home use.

**Table 2:** Distribution of patients with diabetes included in the study.

Patient characteristics	Group 1^a^ (n=11)	Group 2^b^ (n=13)	Group 3^c^ (n=11)	Overall (n=35)
Female, n (%)	5 (45)	4 (31)	1 (9)	10 (29)
Male, n (%)	6 (55)	9 (69)	10 (91)	25 (71)
Age (years), median (range)	50 (37-80)	61 (40-71)	64 (50-73)	62 (37-80)
Age when diagnosed with diabetes (years), median (range)	46 (29-70)	46 (22-61)^d^	46 (32-65)	46 (29-65)
Length of time living with diabetes (years), median (range)	8 (1.5-30)	15 (5-47)^d^	13 (4-40)	11 (1.5-47)
Patients with type 1 diabetes, n (%)	0 (0)	0 (0)	2 (18)	2 (6)
Patients with type 2 diabetes, n (%)	11 (100)	13 (100)	9 (82)	33 (94)

^a^Group 1 included subjects with diabetic peripheral neuropathy (DPN) and no previous history of ulcers.

^b^Group 2 included subjects with DPN and a previous history of ulcers.

^c^Group 3 included subjects with DPN and a current preulcer as determined by the investigator.

^d^Age at diagnosis not available for two subjects.

**Table 3:** Patient responses to statements on the mobile app.

Statements	Response score^a^, mean (SD)	Response score, median (IQR^b^)
It is easy to connect the sock to the mobile app	3.4 (0.89)	3 (0.75)
The mobile app gives useful information about my feet	3.5 (1.10)	4 (1)
The overview of the feet in the mobile app is easy to understand	3.6 (1.04)	4 (1)
I would use the app every day	3.9 (0.91)	4 (2)

^a^Responses were on a 5-point scale where 5 is “Completely Agree” and 1 is “Completely Disagree.”

^a^IQR: interquartile range.

The mobile app was found to be useful and easy to use. The mean and the median responses to key statements on the mobile app are shown in Table 3. Users found it easy to navigate the mobile app and they found the information provided to be informative about their feet.

### Case Studies

A few illustrative cases are shown below, one from each of the three study groups.

#### Case Study 1

**Figure 6:**
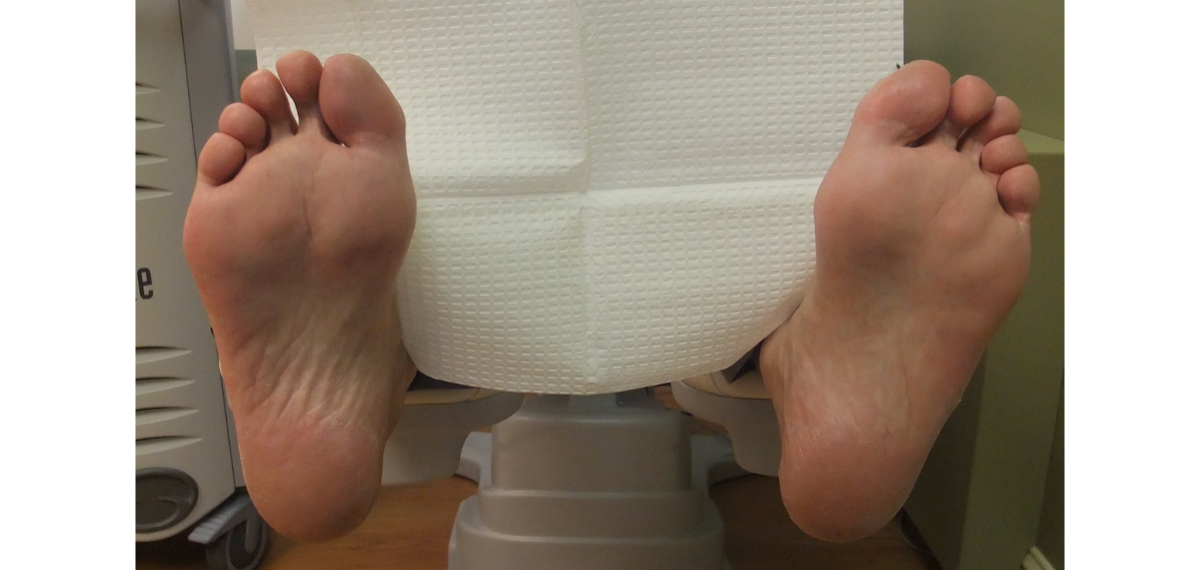
A digital photograph of patient 14’s feet show no visible signs of injury.

**Figure 7:**
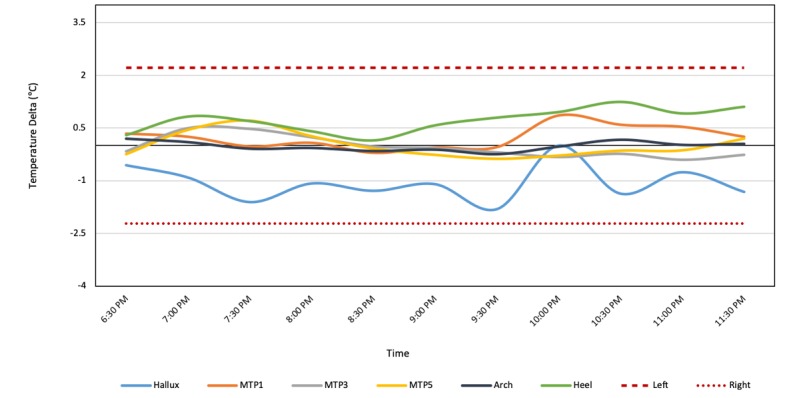
Each line on the graph shows a moving average of the temperature difference (ie, left foot temperature-right foot temperature) for the hallux (blue), metatarsal points 1, 3, and 5 (orange, gray, and yellow, respectively), arch (black), and heel (green). The lines span the period the socks were worn, with time shown on the x-axis. The dashed and dotted red lines show the 2.2°C temperature threshold for the left and right foot, respectively. MTP: metatarsal point.

Patient 14 (Group 1) is a 64-year-old male diagnosed with type 2 diabetes at 53 years of age. He has no history of foot ulceration or amputation and has experienced neuropathic pain for the past 8 years. His feet showed no visible signs of injury (see Figure 6). Patient 14 wore the socks for 6 hours, during which minor variations in temperature between the contralateral locations were observed with differences of less than 2.2°C or 4°F (see Figure 7). The continuous monitoring of the temperature by the socks show minor variations over the 6-hour period. Consistent with the initial observations and medical history, no temperature elevations were found.

#### Case Study 2

**Figure 8:**
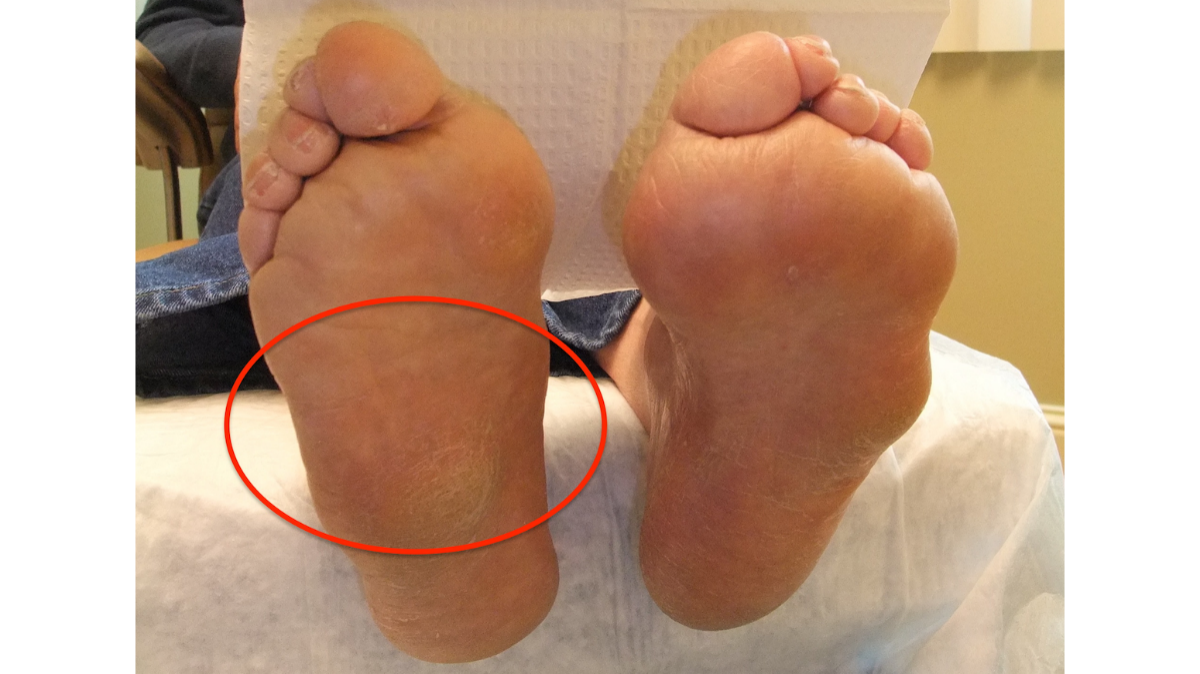
A digital photograph of patient 30’s feet show Charcot of the right foot with collapsed midfoot (arch), designated by the red circle.

**Figure 9:**
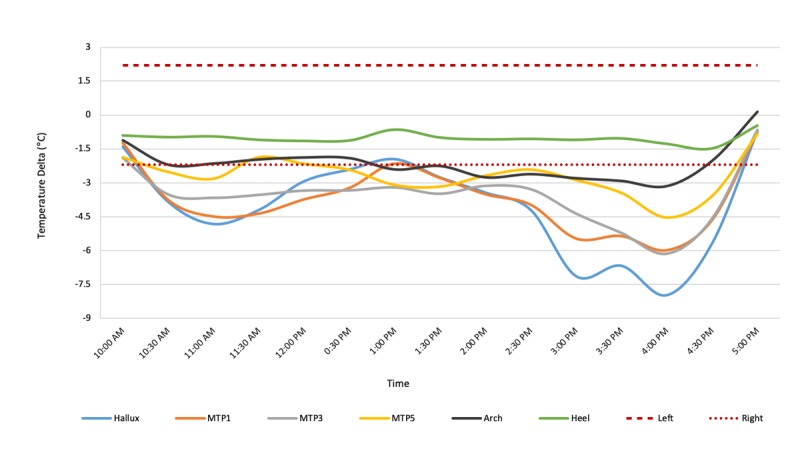
Each line on the graph shows a moving average of the temperature difference (ie, left foot temperature-right foot temperature) for the hallux (blue), metatarsal points 1, 3, and 5 (orange, gray, and yellow, respectively), arch (black), and heel (green). The moving average of the temperature difference shows elevated temperatures of the right foot compared to the left foot at all points except the heel. MTP: metatarsal point.

Patient 30 (Group 2) is a 63-year-old male diagnosed with type 2 diabetes at 45 years of age. He has a history of ulcers and was diagnosed with Charcot arthropathy of the right foot at 57 years of age. Intake photographs (see Figure 8) and examination showed Charcot of the right foot with a collapsed midfoot. Patient 30 wore the sensor-embedded socks for 8 hours, during which period the right foot was consistently warmer than the left foot. The temperatures on the right hallux, MTP 1, MTP 3, MTP 5, and midfoot (arch) were elevated more than 2.2°C or 4°F, up to 8°C (see Figure 9).

Thus, the findings from continuous temperature monitoring are consistent with the patient’s medical history and intake evaluation of Charcot of the right foot. This suggests that the clinical assessment of Charcot arthropathy may benefit from this monitoring system, as it provides a temperature map of the entire foot over a long period of time, rather than static and local temperature changes.

#### Case Study 3

**Figure 10:**
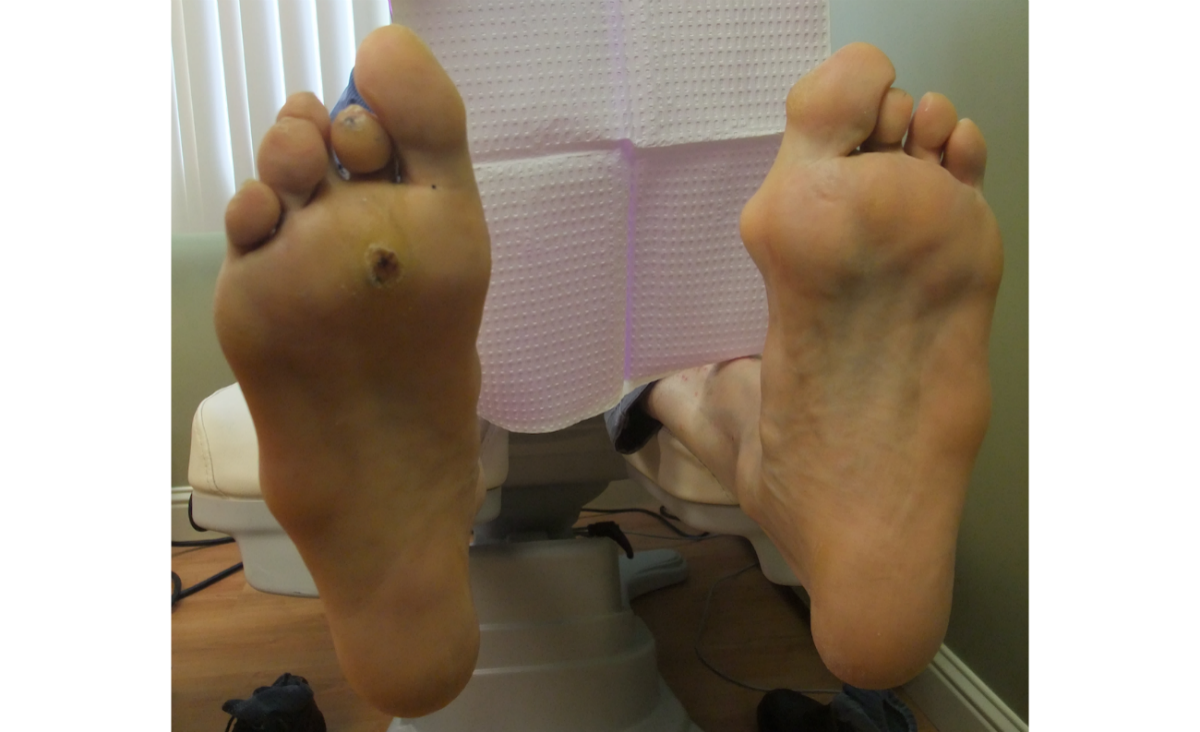
A digital photograph of patient 16’s feet show a preulcerative lesion between the second and third metatarsal.

**Figure 11:**
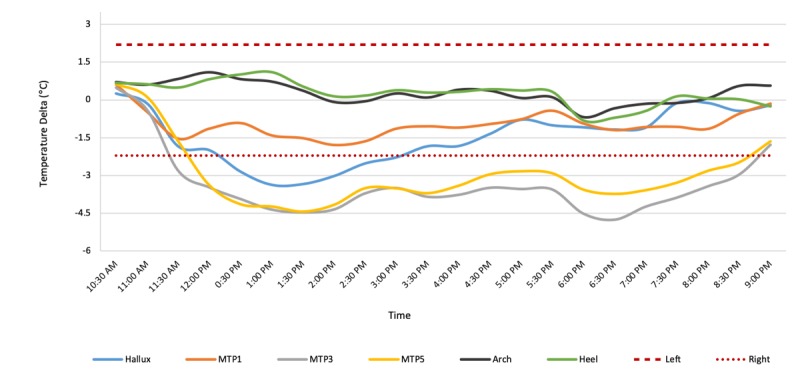
Each line on the graph shows a moving average of the temperature difference (ie, left foot temperature-right foot temperature) for the hallux (blue), metatarsal points 1, 3, and 5 (orange, gray, and yellow, respectively), arch (black), and heel (green). The moving average of the temperature difference shows elevated temperatures at metatarsal points 3 and 5 of the right foot. MTP: metatarsal point.

Patient 16 (Group 3) is a 73-year-old male diagnosed with type 2 diabetes at 65 years of age. He has a history of preulcerative lesions. Intake photographs (see Figure 10) and exam indicated a current preulcerative lesion at the right plantar region between the second and third metatarsal. Patient 16 wore the socks for 9 hours, during which higher temperatures were recorded by two of the six sensors, at the positions of the right metatarsals 3 and 5 (see Figure 11). This observation is consistent with the patient’s medical records indicating a preulcerative lesion in the MTP 2-3 area. These data suggest that in high-risk patients, continuous monitoring may be able to pick up an injury or preulcerative lesion.

## Discussion

### Principal Findings

To our knowledge, this is the first study to introduce wireless continuous temperature monitoring of feet for daily and home use in patients with diabetes. The sensor-embedded socks introduced here contain microsensors embedded directly and seamlessly into the fabric and are designed to look and feel like any other garment. Particularly important for daily and home use, the socks are wireless. Wireless transmission of data is achieved via Bluetooth technology. Through the mobile app, wearers can view the current temperature as measured at six points on the user’s foot. While the app was not set up to generate alerts in this study, users can receive a notification, alert, or text message when a temperature increase is detected between contralateral positions.

The aim of this study was to assess whether these sensor-embedded socks can measure temperature accurately on a continuous basis, whether the temperature findings are consistent with clinical observations, and to obtain feedback on patient experience in using the socks. The temperature studies presented here show that the sensors used in the socks are reliable and accurate at detecting temperature.

In this pilot study of 35 patients, participants found the socks to be no different from standard socks in terms of wearability and reported feelings of comfort and safety. Patients found the app interface to be useful. Furthermore, as shown in the illustrative cases, the temperature differences between the two feet as recorded by the sensor-embedded socks were consistent with the clinical status of the patient.

An earlier report described socks made of optical fiber designed for the clinic environment [28]. A more recent design of smart socks has woven-in sensors and measures temperature at 10-minute intervals, but contains wires [29]. The sensor-embedded socks described in this study are designed for daily wear, both inside and outside the home; are wireless; are machine washable; and do not need to be recharged. Unlike garments made of optical fiber, these socks are made of smart textile, which is designed to be made on standard industrial equipment and can be used anywhere without assistance.

For patients with diabetes and neuropathy, continuous temperature monitoring for the feet now offers information that was not previously available or possible, as was the case with the introduction of continuous glucose-monitoring technology for blood glucose levels.

Static or once-a-day measurements can present a risk of reporting false positives. With continuous monitoring, algorithms can be designed to identify and filter out outliers in measurements spanning several hours and, thus, can potentially reduce false positives by taking into consideration trends over time instead of a single static threshold. As shown in Case Study 2, temperature measurements of the entire foot may be particularly beneficial to patients with Charcot arthropathy. For patients undergoing treatment for an existing injury as in Case Study 3, continuous temperature monitoring provides an objective method to identify injuries.

Patterns of temperature can be obtained via continuous temperature monitoring that are specific to individuals and, in the future, variations from a person’s typical pattern may trigger alerts, rather than a single one-size-fits-all temperature threshold for all individuals. Monitoring patients over time may reveal temporal changes in individual temperature patterns. With further research, algorithms can be developed to detect temperature differences within one foot, without the need for the contralateral foot. Advanced statistical pattern recognition analysis could be used to determine patterns indicative of diabetes-related foot complications.

In future iterations, sensor-embedded socks can be coupled with built-in activity tracking to improve adherence and monitor patient compliance: data from the socks can be used to monitor patient activity and determine whether the patient is compliant with set activity guidelines.

This unique new data stream opens up questions regarding the manner in which the results are best reported, on the content and frequency of notifications, whether preulcerative lesions can be prevented from developing into ulcers, and whether amputations can be reduced. The pilot study reported here was not statistically powered to assess the performance characteristics of this novel device.

Further studies are planned to address such questions, with patient follow-up to obtain data on correlations of the temperature findings with patient outcomes.

### Strengths and Limitations

The strengths of the study are as follows. The sensor-embedded socks were found to work reliably and consistently. The temperature differences reported matched clinical observations. Importantly, the study confirmed that patients can use the socks as a part of their daily lives, within or outside the home. Furthermore, the automatic collection and analysis of the data remove the element of subjectivity from the measurements as currently exists in visual inspection [30].

This study was not without limitations. As it was a single-day study, the findings could not be correlated with longer-term outcomes. More research is needed to further understand data points in continuous temperature monitoring, including as it relates to patient activity and timely intervention. Socks with built-in activity tracking and monitoring are planned to reliably and accurately measure activity concurrent with temperature measurement to further reduce subjective reporting. Future studies will be statistically powered to collect and analyze temperatures and correlate the findings to patient outcomes.

### Conclusions

In this study, we explored the first use of wireless continuous temperature monitoring for daily and home use in patients with diabetes and neuropathy. This noninvasive device designed to behave as a normal sock is the first of its kind to combine wireless continuous temperature monitoring into a wearable device. The socks appear to the wearers to be no different than standard socks. When used with the mobile app, the wearer is kept informed about temperature increases in one foot relative to the other. The socks can reliably and consistently collect temperature data from the wearer’s feet, which are consistent with clinical observations. Continuous temperature monitoring has emerged as a promising tool which could serve as an early warning system for the management of foot ulcers, Charcot foot, and reulceration.

## Abbreviations

DFU: diabetic foot ulcer
DPN: diabetic peripheral neuropathy
IQR: interquartile range
MTP: metatarsal point
